# Determinants and their spatial heterogeneity of carbon emissions in resource-based cities, China

**DOI:** 10.1038/s41598-024-56434-2

**Published:** 2024-03-11

**Authors:** Chenchen Guo, Jianhui Yu

**Affiliations:** 1 Institute of Geographic Sciences and Natural Resources Research, CAS, Beijing, 100101 China; 2 College of Resources and Environment, University of Chinese Academy of Sciences, Beijing, 100049 China; 3 CAS Key Laboratory of Regional Sustainable Development Modeling, Beijing, China

**Keywords:** Carbon emissions, Spatial heterogeneity, Driving factors, Geographically weighted regression model, Resource-based city, Climate-change impacts, Climate-change mitigation, Climate-change adaptation, Climate-change impacts, Climate-change mitigation, Climate-change policy, Energy and society, Environmental economics, Environmental impact, Socioeconomic scenarios, Sustainability

## Abstract

Global climate change associated with increased carbon emissions has become a global concern. Resource-based cities, by estimations, have emerged as major contributors to carbon emissions, accounting for approximately one-third of the national total. This underscores their pivotal role in the pursuit of carbon neutrality goals. Despite this, resource-based cities have long been neglected in current climate change mitigation policy discussions. Accordingly, using exploratory spatial data analysis and Geographical Weighted Regression method, this study investigates the determinants of carbon emissions and their spatial pattern in 113 resource-based cities in China. It can be concluded that: (1) The proportion of carbon emissions from resource-based cities in the national total has shown a marginal increase between 2003 and 2017, and the emissions from these cities have not yet reached their peak. (2) A relatively stable spatial pattern of “northeast high, southwest low” characterizes carbon emissions in resource-based cities, displaying significant spatial autocorrelation. (3) Population size, economic development level, carbon abatement technology, and the proportion of resource-based industries all contribute to the increase in carbon emissions in these cities, with carbon abatement technology playing a predominant role. (4) There is a spatial variation in the strength of the effects of the various influences.

## Introduction

Climate change, stemming from heightened carbon emissions, presents substantial implications for economic growth and social development, making it a global concern^1^. China's energy consumption has generally exhibited a high-rise trend due to the steady progress of urbanization and industrialization, which has provoked considerable international attention^2,3^. As stated by the International Energy Agency, surpassing the United States in 2007, China has become the top emitter in the world, responsible for about one-third of the world's total carbon emissions. Faced with escalating international pressure for carbon emissions reduction, China is confronted with significant challenges to reduce its carbon footprint. In response to this, China has pledged to peaking its carbon emissions by 2030 and reaching carbon neutrality by 2060.

Resource-based cities (RBCs) are cities whose primary economic activity is the extraction and processing of local natural resources^4,5^. Endowed with abundant natural resources, these cities have long played the role of resource and energy supply base for the region and even the country^6–8^. However, according to the "Carbon Curse" theory, fossil fuel endowment is highly related to high carbon emissions^9^. Under comparable conditions, carbon-intensive development is more likely to occur in regions with abundant fossil fuel resources. Estimations indicate that RBCs have become the region with the largest carbon emissions^10^, emitting around one-third of the total national carbon emissions. In this context, the low-carbon transformation of RBCs is of critical significance in terms of achieving the “30/60” dual carbon goal. Despite their crucial role, current discussions on climate change policies have consistently overlooked resource-rich regions^9^. While a great deal of attention has been paid to the differences between established and emerging economies, the differences between resource-rich regions and resource-poor regions have been neglected. Further, the distinctive social and economic circumstances of resource-rich regions, such as RBCs, pose challenges for the effective implementation of carbon reduction policies designed for more conventional regions. Therefore, resource-rich regions, such as RBCs, require special attention. Studying the factors that affect carbon emissions in RBCs can help better understand resource-rich regions and the barriers they must overcome in carbon reduction work. By taking these steps, we will be able to direct the climate debate in a more constructive direction and better appreciate the challenges inherent in global carbon reduction.

Population, economy, technology, and industry all contribute to urban carbon emissions. In the 1990s, Engelman^11^ observed a parallel growth rate between global population and carbon emissions, sparking the hypothesis that population growth is a key factor driving the increase in global carbon emissions. Since then, increasing numbers of scholars have examined the relationship between population and carbon emissions^12,13^, finding that the expansion of population size results in an increase in carbon emissions by expanding production and consumption activities^14^. The discourse on the relationship between economic development and environmental protection has persisted for years. Because economic entities around the world rely heavily on fossil fuels to promote economic growth and meet the increasing demand for population growth^15^, economic growth has become one of the contributing factors to the rise in carbon emissions^16,17^. However, the relationship will revert once economic development arrives at a certain level, due to advancements in technology and industrial structure^18,19^. Technological innovation is considered paramount in reducing carbon emissions^20–22^. The innovation of related new energy, materials and processes can effectively reduce the carbon emissions of the energy industry in the production process^23–26^. However, the impact of technology on carbon emissions has both a direct and scale effect^27^. The enhancement of energy utilization efficiency enables innovative technologies to negatively influence carbon emissions^28^. This is referred to as the “direct effect”. Meanwhile, technological innovation can also promote the growth of economic scale and output level, increasing energy consumption and finally causing the rise of carbon emissions^29^, which is the “scale effect”. Industrial development stands as another factor influencing carbon emissions^30^. Multiple studies have explored the significance of various industrial sectors, such as the transport sector^31^, the garment sector^32^, and the building sector ^33^, on carbon emissions. Although several studies have explored the impact of mining on carbon emissions, most have focused on the influence of coal obtained from mining as an energy source rather than the coal mining processes themselves. Furthermore, even less attention has been paid to the impact of the dependence of urban economic development on the mining industry on urban carbon emissions.

Following the above analysis, we propose to address the following three questions: (1) What constitutes the spatial pattern of carbon emissions in RBCs, and does this pattern exhibit temporal variations? (2) In what manner do population, economy, technology, and industry affect the carbon emissions of RBCs? (3) What spatial pattern characterizes the impacts of these factors, and does this pattern undergo alterations over time?

Based on these questions, this study aims to have the following four objectives and contributions: (1) This paper supplements the research gap in the existing study by including resource-rich regions in the field of carbon emissions for the first time. (2) Incorporating spatial heterogeneity and spatial dependence into the classical linear model, this study deeply explores how various factors affect Chinese RBCs’ carbon emissions, what spatial patterns their intensity exhibit and what are the reasons for formation. (3) By including the proportion of resource-based industries variables as potential drivers of carbon emissions, we examine the impact of the dependence of urban economic development on the mining industry on urban carbon emissions for the first time. Even though several studies have revealed that industrial structure can have a direct or indirect effect on carbon emissions, few studies have incorporated resource-based industries into their analyses. It is the first time that the dependence of urban economic development on resource-based industries is taken as an influencing factor.

The remaining part of this paper is organized as follows. Section “Literature review” is a literature review. The methodology and data are presented in Section “Methodology”. Section “Empirical analysis” provides a detailed analysis of the spatial and temporal patterns of carbon emissions from China’s RBCs as well as their influencing factors. Section “Conclusions” offers conclusions of the research and proposes possible extensions for future research.

## Literature review

With the introduction of the carbon emission reduction target, carbon emissions have become a hot topic in research. Current studies cover various aspects, including the calculation of total amount^34^, footprint^35^, intensity^36,37^, and carbon efficiency performance^38,39^, as well as the investigation of factors affecting them^40,41^ and the spatial and temporal patterns of change^42,43^.

Over the past several years, increasing attention has been drawn to the issue of carbon emissions and the influence they exert on the environment. This has led to a surge in research efforts aimed at understanding the spatial distribution of carbon emissions across different regions and countries. Overall, researchers have primarily focused on studying the spatial difference in carbon emissions by using administrative units such as countries, regions, provinces, cities, and counties. Studies at the country level have revealed that the Asia–Pacific Rim region exhibits dense carbon emissions^44^. Furthermore, the difference between national groups has been identified as the main reason for the formation of the differences in the intensity of carbon emissions across countries^45^. At the regional level, studies have demonstrated noteworthy spatial disparities in China's carbon emissions^46,47^. However, the source of these differences, whether they arise within or between the four major regions, remains undefined^48–50^. Similar findings have been reported at the provincial level^51–53^. China's carbon emissions show a pattern of "lower in the south and east and higher in the north and west"^52^, with high-value regions located in the eastern and southern provinces^54^. However, the difference in carbon emissions between provinces appears to be decreasing^51,52^. This conclusion has also been confirmed in research at the municipal scale^37,41,42,55^. As township-level data becomes crucial for understanding China's economic growth, recent research priorities have shifted towards this level. Some studies have observed a substantial spatial polarization effect of per-capita carbon emissions at the township level, together with significant spatial autocorrelation and a spatial distribution characterized by "high in the north and low in the south"^56^. In mainstream academic research, various methods are commonly used, including standard deviation ellipse, Theil indices, Gini coefficient, Atkinson index, variance, variance coefficient, convergence theory, and spatial autocorrelation model.

The factors affecting carbon emissions are diverse and complex. Researchers have engaged in the investigation of these factors using various methodologies, including the IPAT model, STIRPAT model, input–output analysis, structural decomposition analysis, LMDI factor decomposition analysis, and geographical detector.

At the national scale, Xv et al.^57^ examined factors influencing China's carbon emissions using the LMDI method. Their research revealed that carbon emissions are negatively impacted by energy structure and energy efficiency while being positively influenced by economic growth. However, while improvements in energy efficiency and energy structure can reduce carbon emissions, they cannot counterbalance the rise in carbon emissions resulting from economic development^57^. Sadorsky et al.^58^ discovered that urbanization can both positively and negatively impact carbon emissions, which often complement one another. Accordingly, urbanization barely affects carbon emissions on a statistical basis^58^. Taking countries in the Association of Southeast Asian Nations (ASEAN) as examples, Chontanawat et al.^59^ utilized the extended IPAT model to decompose carbon emissions and discovered that population and economic growth are the key players in the surge of carbon emissions observed in these countries, while improvements in energy efficiency and reduced carbon intensity serve as a restraint on carbon emissions growth^59^. Cheng et al.^60^ researched 35 OECD countries using a panel data quantile regression model. The results suggested that technological innovation can significantly mitigate the rise of carbon emissions associated with economic growth, confirming the Environmental Kuznets Curve (EKC) hypothesis^60^.

At the provincial scale, Lantz et al.^61^ selected five Canadian regions as case sites. Through analyzing data from 1970 to 2000, they confirmed an inverted U-shaped relationship between population size and carbon emissions^61^. In a related study by Cheng et al.^62^, the spatiotemporal patterns of China's energy consumption and carbon emissions intensity are found to be influenced by several factors, including energy intensity, energy structure, industrial structure, and urbanization rate. To examine how social and economic factors affect carbon emissions at the provincial level in China, Li et al.^63^ utilized the Geographically Weighted Regression (GWR) model in their research, finding that carbon emissions intensity, per capita GDP, and per capita total social investment are all contributors to per capita carbon emissions. A related study by Zhang et al.^64^ found that urbanization impacts carbon emissions either positively or negatively based on the region, which is further confirmed in the research conducted by Wang et al.^65^. The complexity of the relationship between urbanization and carbon emissions can be attributed to the fact that the type of energy used in industry and housing has a great effect on urbanization's impact on carbon emissions^64,65^.

At the municipal scale, a geographical detector was used by Wang et al.^10^ to examine the driving factors of carbon emissions in China and determined that energy intensity is the dominant factor that contributes to spatial heterogeneity in carbon emissions among RBCs and northern cities. The urban economic size of non-resource-based cities and southern cities plays a substantial role in the spatial heterogeneity of carbon emissions^10^. A study conducted by Wang et al.^42^ demonstrated that economic growth, population expansion, industrial structure, and capital investment all positively influence per capita carbon emissions in Chinese cities and that per capita carbon emissions are correlated with economic development in an inverted U-shape.

In recent years, academic research has gradually shifted its focus to the municipal scale. Wang et al.^56^ expanded the STIRPAT theoretical framework and introduced the EKC hypothesis into their research, using a panel quantile regression model to explore how social and economic factors, such as population density, government public expenditure, and the size of the second industry output, influence per capita carbon emissions at the municipal scale. They confirmed that population density and government public expenditure have a suppressive effect on per capita carbon emissions, while the size of the second industry output has a promoting effect^56^. Overall, carbon emissions are profoundly impacted by population size, the level of economic development, energy consumption, industrial structure, and urbanization level. However, these factors interact in a complicated manner, and their impacts on carbon emissions vary across scales, geographic regions, and time.

Generally speaking, researchers have contributed significantly to the field of carbon emissions, laying the groundwork for this article. However, existing studies generally focus on the investigation of geographic units at various scales, including national, regional, provincial, city, and town levels, but have not yet addressed special types of cities, such as resource-rich regions.

## Methodology

### Research area

**Figure 1:**
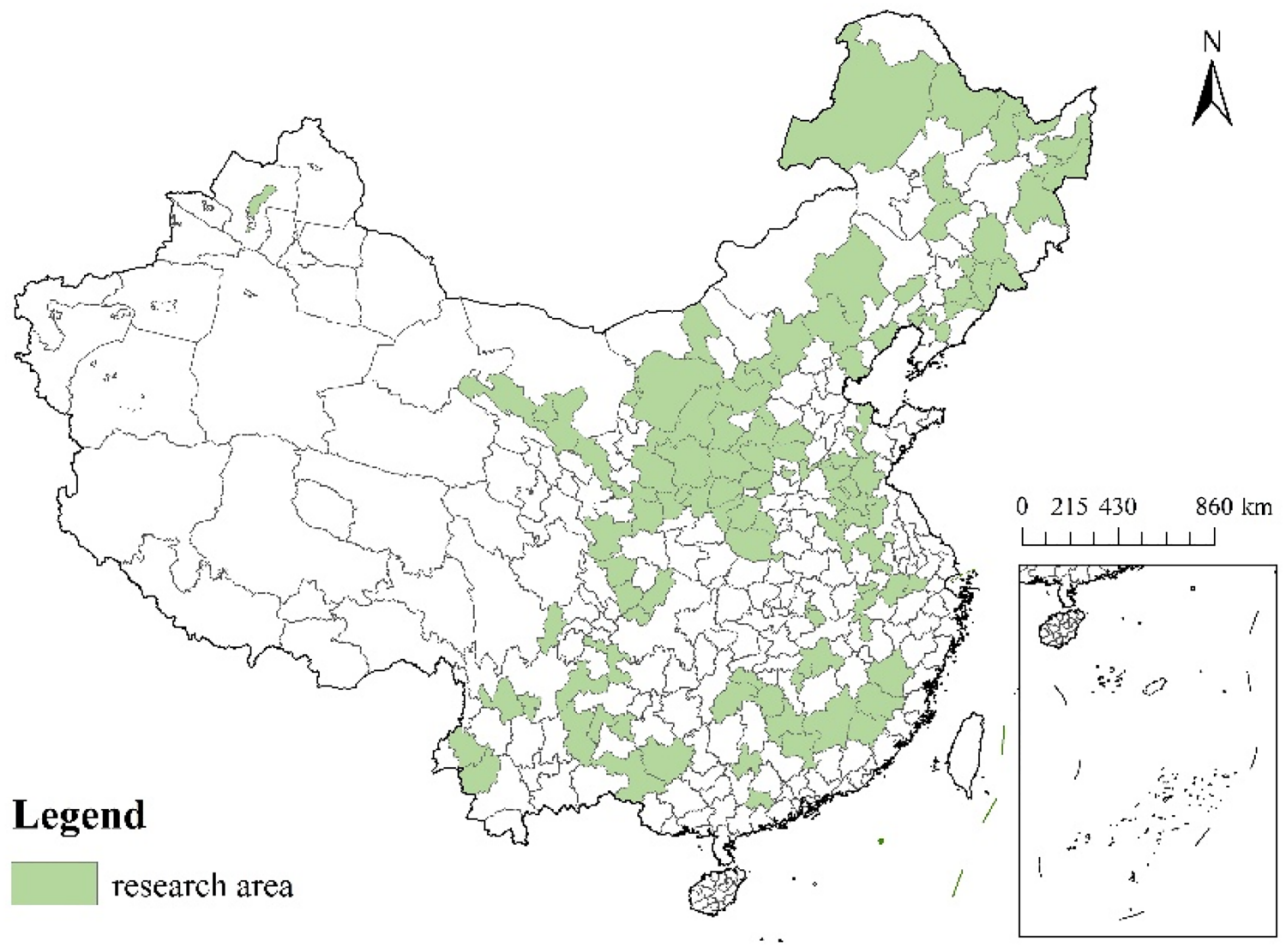
Study area map (source: GS(2019)1822).

Resource-based cities, which take natural resources mining and processing as leading industries, play a critical role in securing China's strategic energy supply. As stated by the National Sustainable Development Plan for Resource-based Cities (2013–2020), China has 262 RBCs. These include 126 prefectures of the People's Republic of China (prefecture-level cities, regions, autonomous prefectures, and unions), 62 county-level cities, 58 counties (autonomous counties, forest regions, etc.), and 16 municipal districts (development zones, management zones). Following the study purpose and data availability, 113 prefecture-level cities were chosen for the study (the remaining cities have severe data deficiencies). The following is an overview map of the research area (see Fig. 1).

### Research methods

#### Exploratory spatial data analysis

Spatial data autocorrelation characteristics can be analyzed by exploratory spatial data analysis, which incorporates adjacent variables and associates specific variables with their location. In this study, we adopted Moran’s I index to examine the spatial autocorrelation of carbon emissions in China’s RBCs. Equation (1) shows the formula:

1 $$\begin{array}{*{20}c} {\begin{array}{*{20}c} {I = \mathop \sum \limits_{i = 1}^{n} \mathop \sum \limits_{j = 1}^{n} w_{ij} \left( {x_{i} - \overline{x}} \right)\left( {x_{j} - \overline{x}} \right)/\mathop \sum \limits_{i = 1}^{n} \mathop \sum \limits_{j = 1}^{n} w_{ij} \mathop \sum \limits_{i = 1}^{n} \left( {x_{i} - \overline{x}} \right)^{2} } \\ \end{array} } \\ \end{array}$$

where $I$ denotes global Moran’s $I$; ${x}_{i}$ and ${x}_{j}$ denote observed carbon emissions in city $i$ and city $j$, respectively; ${w}_{ij}$ represents the spatial weight matrix; $n$ is defined as the number of samples. The range of Moran’s $I$ value is -1 to 1. If the value of Moran’s $I$ exceeds 0, there is a positive spatial correlation. In this context, spatial clustering is more significant at higher values. When the value of Moran’s $I$ is smaller than 0, there is a negative spatial autocorrelation, and a smaller value indicates a greater degree of spatial dispersion. Otherwise, the spatial correlation effect does not exist when the value is 0.

However, the global Moran’s $I$ index only takes into account the overall characteristics of clustering, ignoring the spatial relationships between neighboring regions on a local scale. Therefore, Moran’s $I$ index and Moran scatter plot were used to reveal the spatial clustering characteristics of carbon emissions in local areas. The formula is shown in Eq. (2):

2 $$\begin{array}{*{20}c} {\begin{array}{*{20}c} {I_{i} = n\left( {x_{i} - \overline{x}} \right)\mathop \sum \limits_{j} w_{ij} \left( {x_{j} - \overline{x}} \right)/\mathop \sum \limits_{i = 1}^{n} \left( {x_{i} - \overline{x}} \right)^{2} } \\ \end{array} } \\ \end{array}$$

where ${I}_{i}$ denotes local Moran’s $I$; ${x}_{i}$ and ${x}_{j}$ denote observed carbon emissions in city $i$ and city $j$, respectively; ${w}_{ij}$ represents the spatial weight matrix; $n$ is the number of samples.

#### Geographically weighted regression (GWR) model

Given that the geographical coordinates of the data are integrated into the regression parameters of a GWR model, it is particularly suitable for analyzing spatial phenomena with spatial non-stationarity. A variation of the parameters of the model is facilitated when local geographical coordinates are changed, which allows the spatial relationship between observations of carbon dioxide emissions and the factors influencing them to be revealed. The GWR model can be shown in Eq. (3):

3 $$\begin{array}{*{20}c} {\begin{array}{*{20}c} {y_{i} = \beta_{0} \left( {\mu_{i} ,v_{i} } \right) + \mathop \sum \limits_{k} \beta_{k} \left( {\mu_{i} ,v_{i} } \right)x_{ik} + \varepsilon_{i} } \\ \end{array} } \\ \end{array}$$

where ${I}_{i}$ denotes local Moran’s $I$; ${x}_{i}$ and ${x}_{j}$ denote observed carbon emissions in city $i$ and city $j$, respectively; ${w}_{ij}$ represents the spatial weight matrix; $n$ represents the number of samples.

### Data resource

The prefecture-level carbon emissions data spanning from 1997 to 2017 were compiled by consolidating county-level data extracted from the CEADs database^34^, which calculates emissions based on the consumption principle. Data on the permanent population, per capita gross domestic product, the number of miners, and the number of workers in the first, second, and third industries were compiled from China's Urban Statistical Yearbook. In addition, Statistical Bulletins on National Economic and Social Development from various cities were adopted to supplement the missing data. Further, to eliminate the effect of price factors, the normal per-capita GDP data were converted into constant prices in 2003 using the GDP deflator index.

## Empirical analysis

### An analysis of Chinese RBCs’ spatial and temporal characteristics of carbon emissions

#### Time evolution characteristics

**Figure 2:**
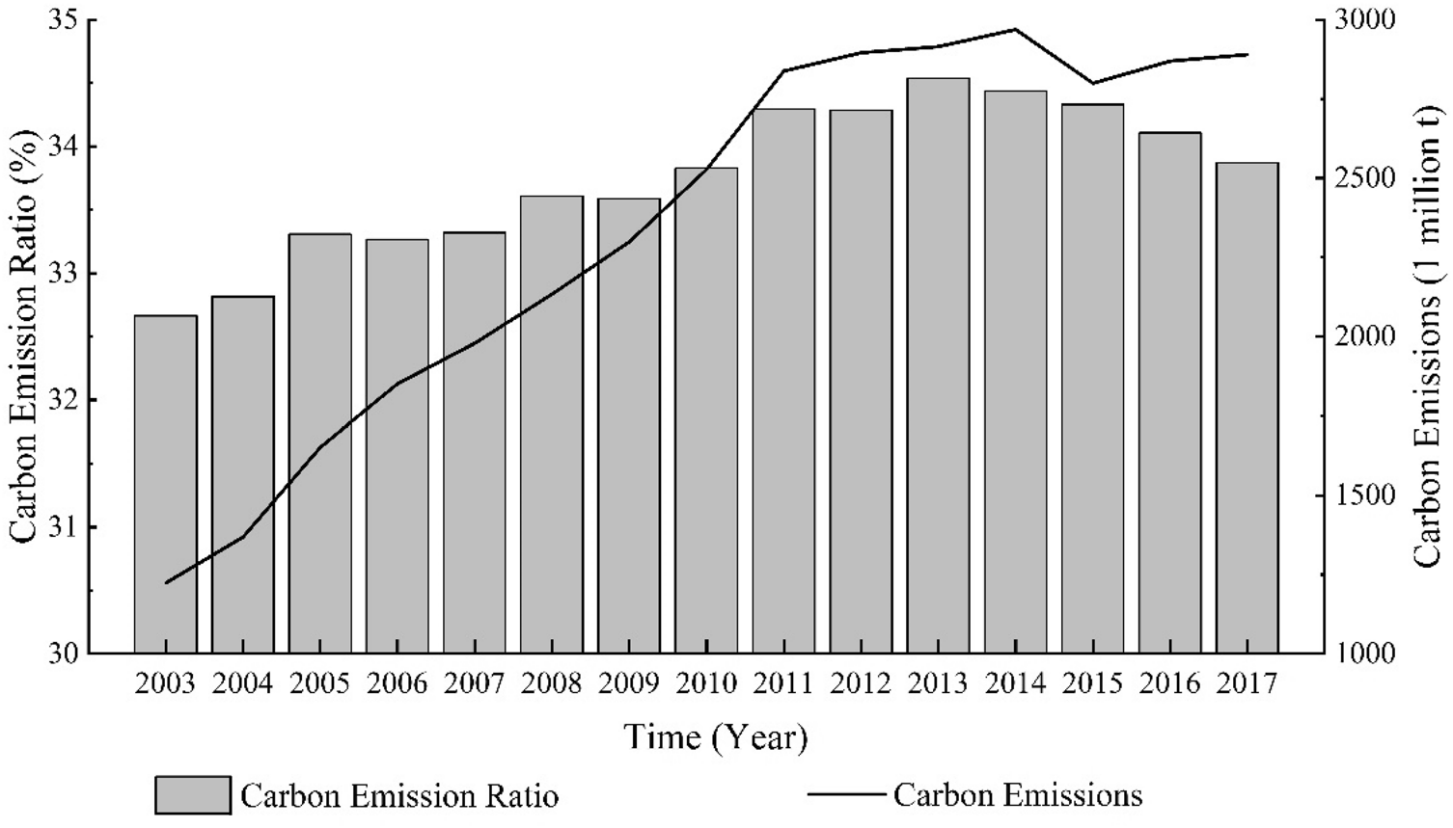
China RBCs' carbon emissions and carbon emission ratio from 2003 to 2017.

From 2003 to 2017, the total carbon emissions from RBCs increased from 1223.49 × 10^6^t to 2889.81 × 10^6^t, with their proportion in total carbon emissions slightly rising from 32.66% to 33.87%. There has been a fluctuating upward trend in total carbon emissions of RBCs, with a total growth rate of 136.19% and an annual growth rate of 6.53%. Specifically, the growth rate of carbon emissions of RBCs showed a characteristic shift from high levels before 2011 to lower levels thereafter (see Fig. 2). From 2003 to 2011, RBCs’ carbon emissions increased rapidly, expanding at an average annual rate of 11.16 percent. However, since 2011, there has been a noticeable deceleration in carbon emissions, with an average growth rate of merely 0.34% annually. The year 2011 marked the introduction of the Twelfth Five-Year Plan on Energy Conservation and Emission Reduction was issued. This plan, aimed at reducing greenhouse gas emissions, established pilot projects for carbon emissions trading and implemented mechanisms for voluntary carbon reduction. These initiatives likely contributed to the significant slowdown in the rise of carbon emissions from RBCs since 2011.

**Figure 3:**
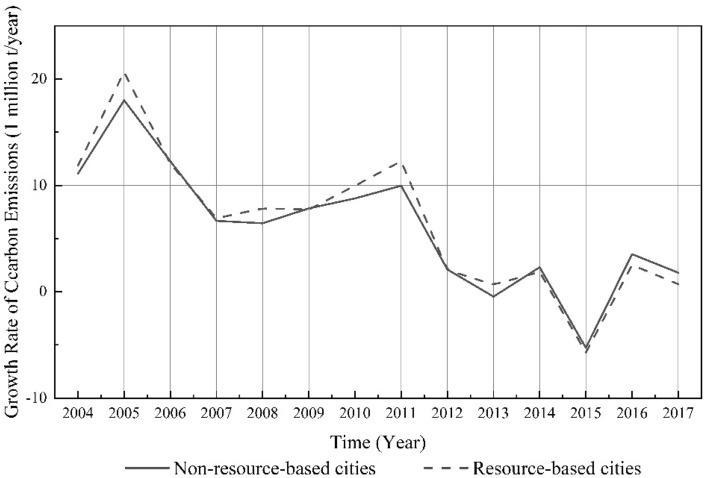
Trends in the growth rate of average carbon emissions from China’s resource-based and non-resource-based cities from 2003 to 2017.

In comparison with non-resource-based cities, RBCs experienced higher growth from 2003 to 2013, but a lower growth rate from 2014 to 2017 (see Fig. 3). This suggests that, after 2013, within the national framework for reducing carbon emissions, RBCs have exhibited a greater decrease in their carbon emissions and garnered superior carbon reduction outcomes than non-resource-based cities. In the Sustainable Development Plan for National Resource-Based Cities (2013–2020), the State Council classifies RBCs into four types based on their resource availability and sustainable development capabilities. According to the plan, each type of city has a specific development direction and important tasks, and various cities will be guided to explore unique development models that reflect their particular strengths. This marked the official launch of the transformation of RBCs in China. The transformation of economic development models in RBCs is a possible explanation for the greater reduction in their carbon emissions after 2013 compared to non-resource-based cities.

As illustrated in Fig. 2, RBCs’ carbon emissions entered a plateau in 2011 and peaked in 2014. To test whether RBCs have achieved “Peak Carbon Dioxide Emissions”, a Mann–Kendall trend test was utilized to estimate the tendency of carbon emissions over time. The results are presented in Table 1. The Z value is significantly greater than 0, whereas the P value is 3.23 × 10^–5^. In light of this, we can conclude that the carbon emissions of RBCs in China have not yet peaked and that further increases are still possible.

**Table 1:** Analysis results of Mann–Kendall trend test.

Area	S	V(S)	Zmk	P	Trend
Resource-based cities	85.00	408.33	4.16	3.23 × 10^–5^	Upward trend

**Figure 4:**
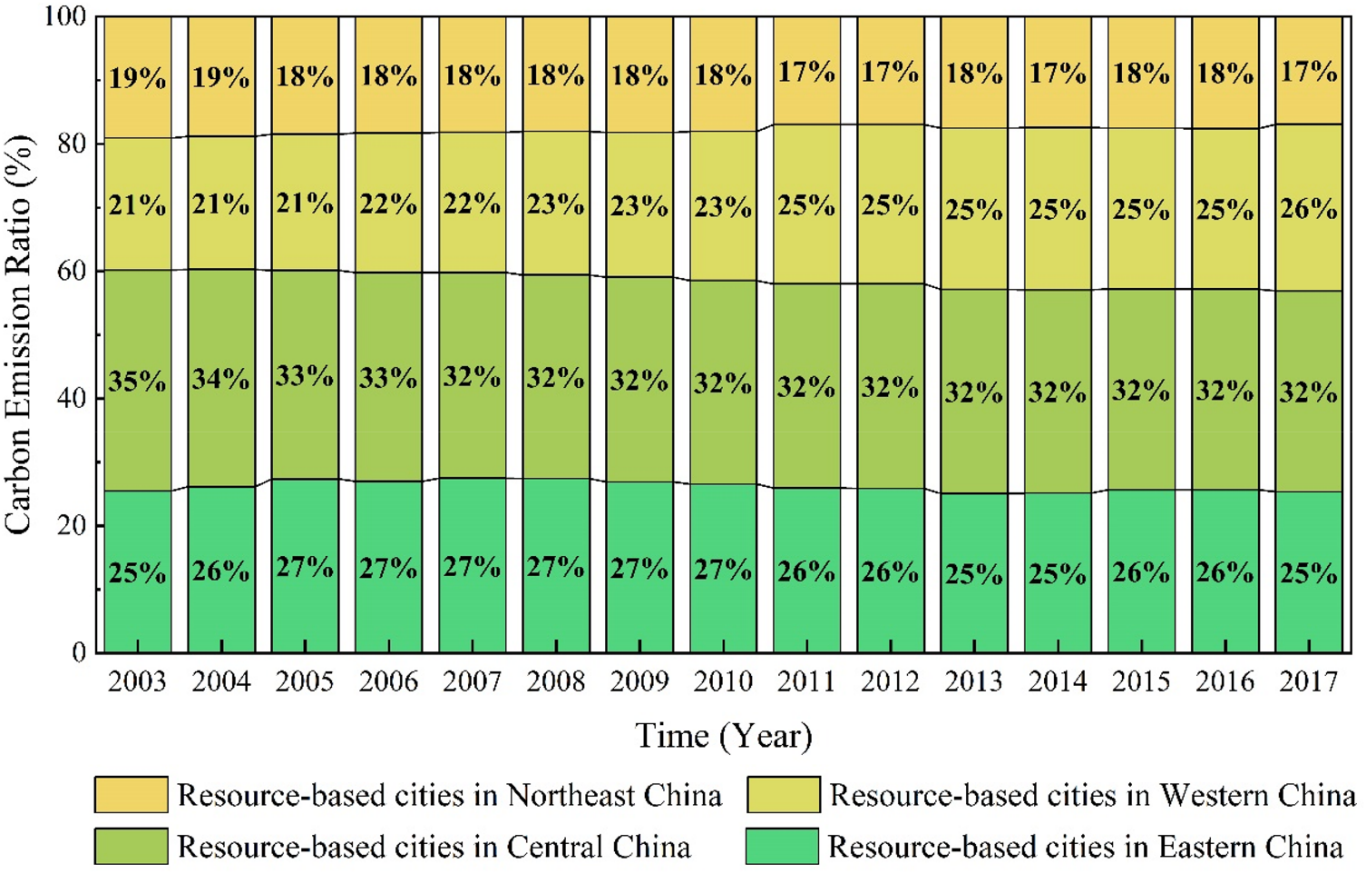
Carbon emissions from RBCs in the four economic regions of China between 2003 and 2017.

RBCs in Central China were the largest emitters, comprising a substantial proportion of overall carbon emissions from RBCs (see Fig. 4). Despite this, carbon emissions from these cities have decreased slightly in recent years, from 35% in 2003 to 32% in 2017. RBCs in Northeast China accounted for the lowest share of carbon emissions, and this share has decreased over time, from 19% in 2003 to 17% in 2017. This may be related to the decline in traditional industries in Northeast China^30^. Moreover, RBCs in Eastern China produced relatively stable carbon emissions during the period 2003–2017. Nevertheless, the share of carbon emissions from RBCs in Western China increased from 21 to 26%. As a whole, RBCs in Central and Western China emitted significantly more carbon dioxide than RBCs in Eastern and Northeast China combined, with their contribution to total RBCs’ carbon emissions in China increasing over time.

**Figure 5:**
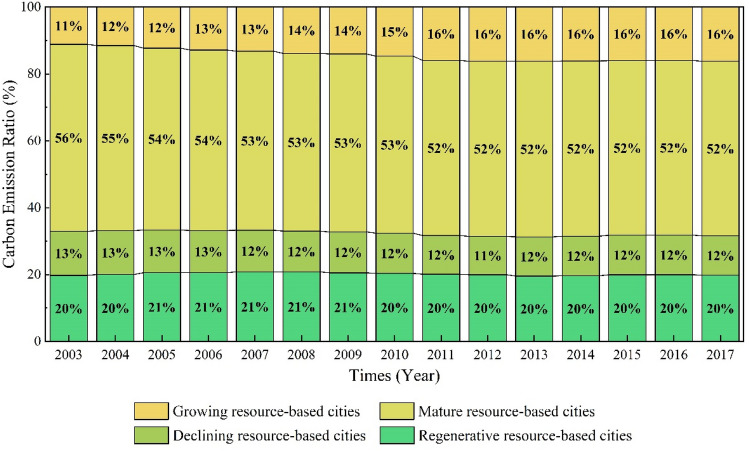
Trends of carbon emissions ratio in growing, mature, declining and regenerative RBCs from 2003 to 2017.

Over the period 2003 to 2017, mature RBCs accounted for the majority of carbon emissions, exceeding 50% (see Fig. 5). However, recently, the share of carbon emissions from mature RBCs has decreased, from 56% in 2003 to 52% in 2017. The share of carbon emissions generated by growing RBCs was the lowest, but it has shown a gradually increasing trend in recent years and has surpassed declining RBCs in 2007 to become the third leading source of carbon emissions among RBCs. The combined proportion of carbon emissions in regenerative RBCs and declining RBCs were relatively stable, ranging from 20 to 22% and 12% to 14%, respectively.

#### Spatial association characteristics

**Figure 6:**
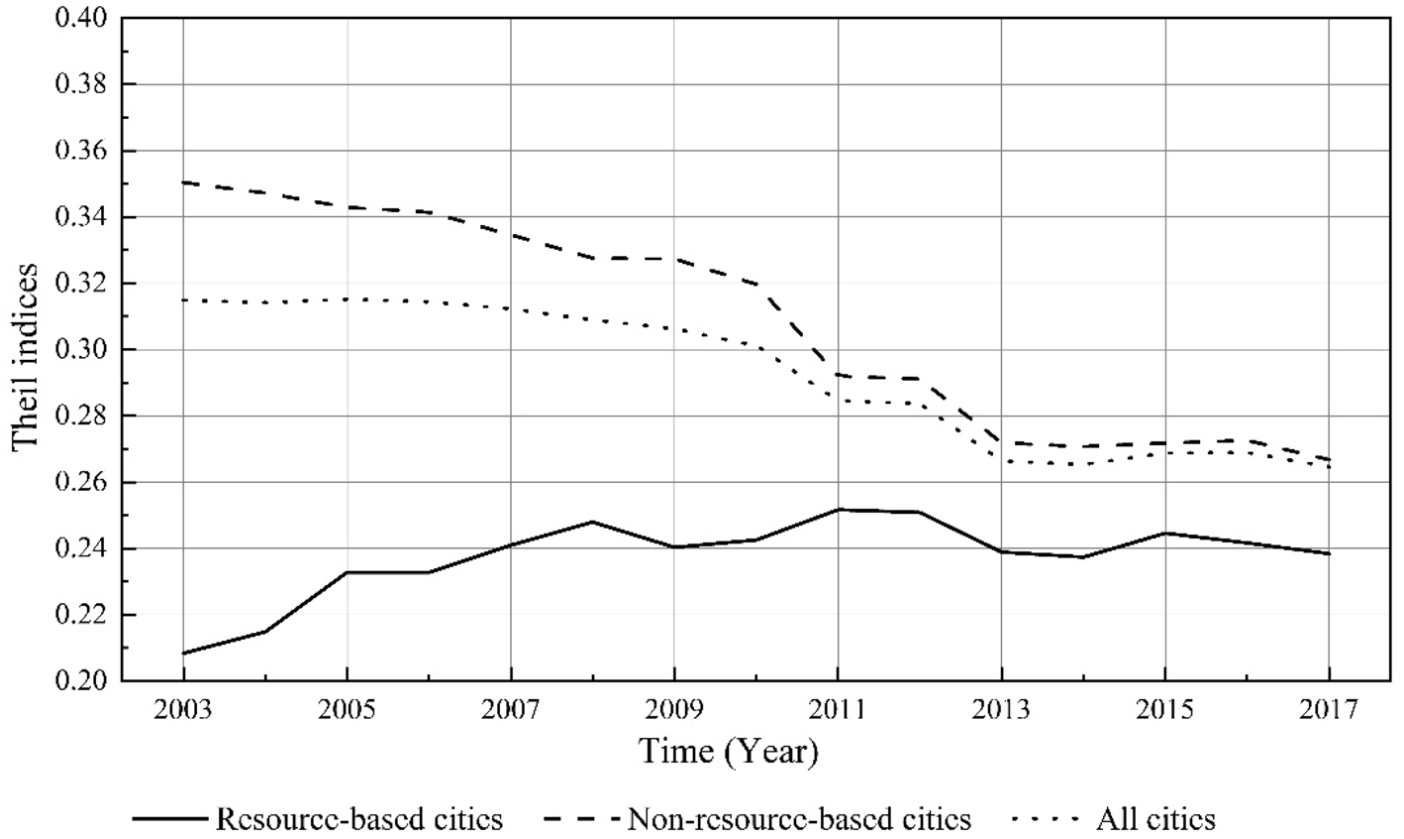
Inter-city differences of carbon emissions among resource-based and non-resource-based cities from 2003 to 2017.

We utilized Theil indices to investigate the spatial disparities in carbon emissions across RBCs. Results demonstrate (see Fig. 6) that the Theil indices of carbon emissions among RBCs were significantly below the national average and also below those of non-resource-based cities. However, contrary to the trend of non-resource-based cities, there has been a gradual increase in the differences in carbon emissions among RBCs from 2003 to 2017.

**Figure 7:**
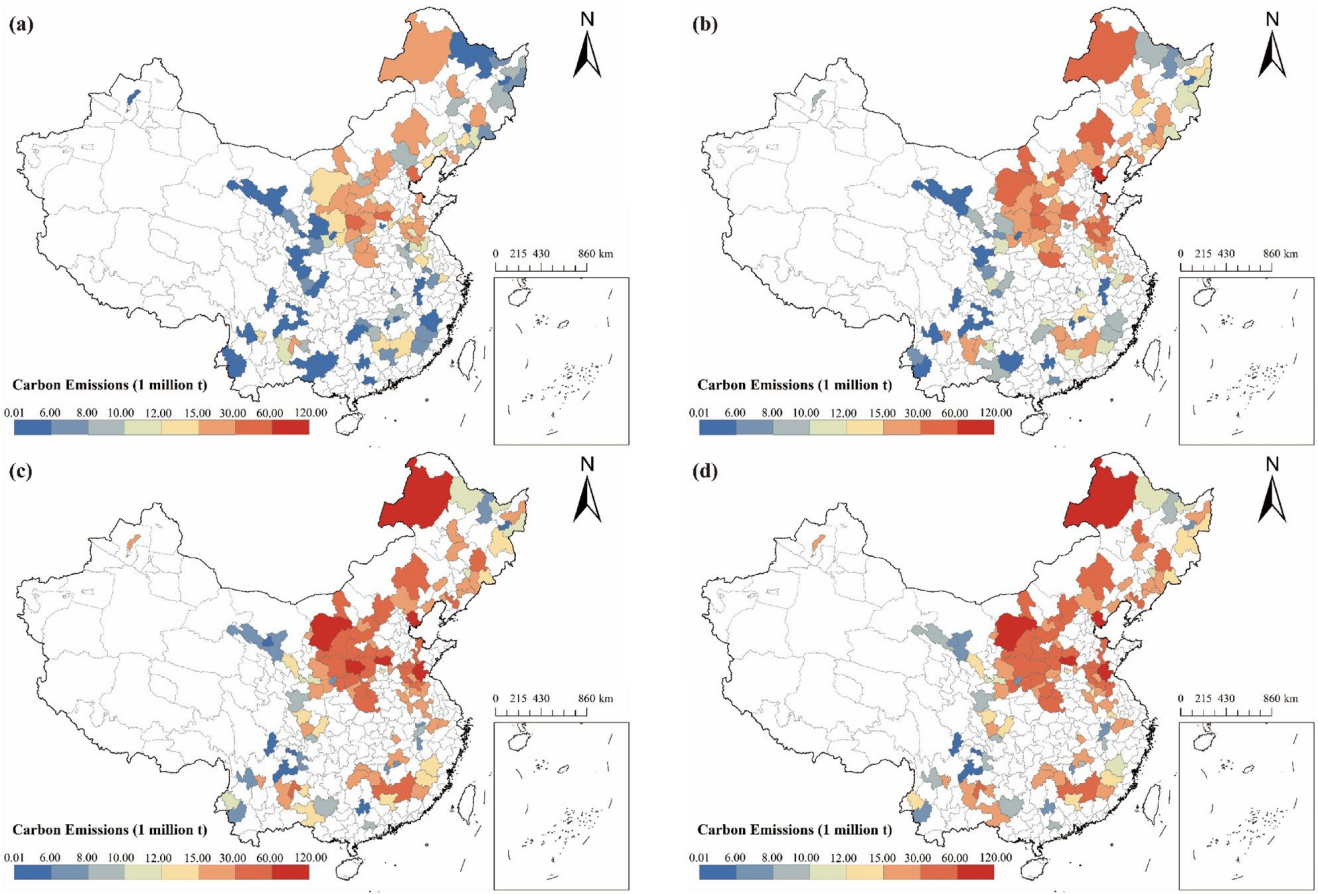
Characteristics of the spatial and temporal distribution of carbon emissions in Chinese RBCs from 2003 to 2017: (**a**) Result in 2003; (**b**) Result in 2007; (**c**) Result in 2012; (**d**) Result in 2017 (source: GS(2019)1822).

A spatial characteristic of "high emissions in the northeast and low emissions in the southwest" was observed in China's RBCs, with the highest values concentrated in Inner Mongolia, Shaanxi, Shanxi, and the northeast region of the country (see Fig. 7). All RBCs have exhibited a rise in carbon emissions over time, with inner Mongolia, Shaanxi, Shanxi, and the three northeastern provinces displaying a particularly prominent trend. This is related to the increasing activities of extraction and processing of coal, oil, and gas in these resource-rich regions.

**Figure 8:**
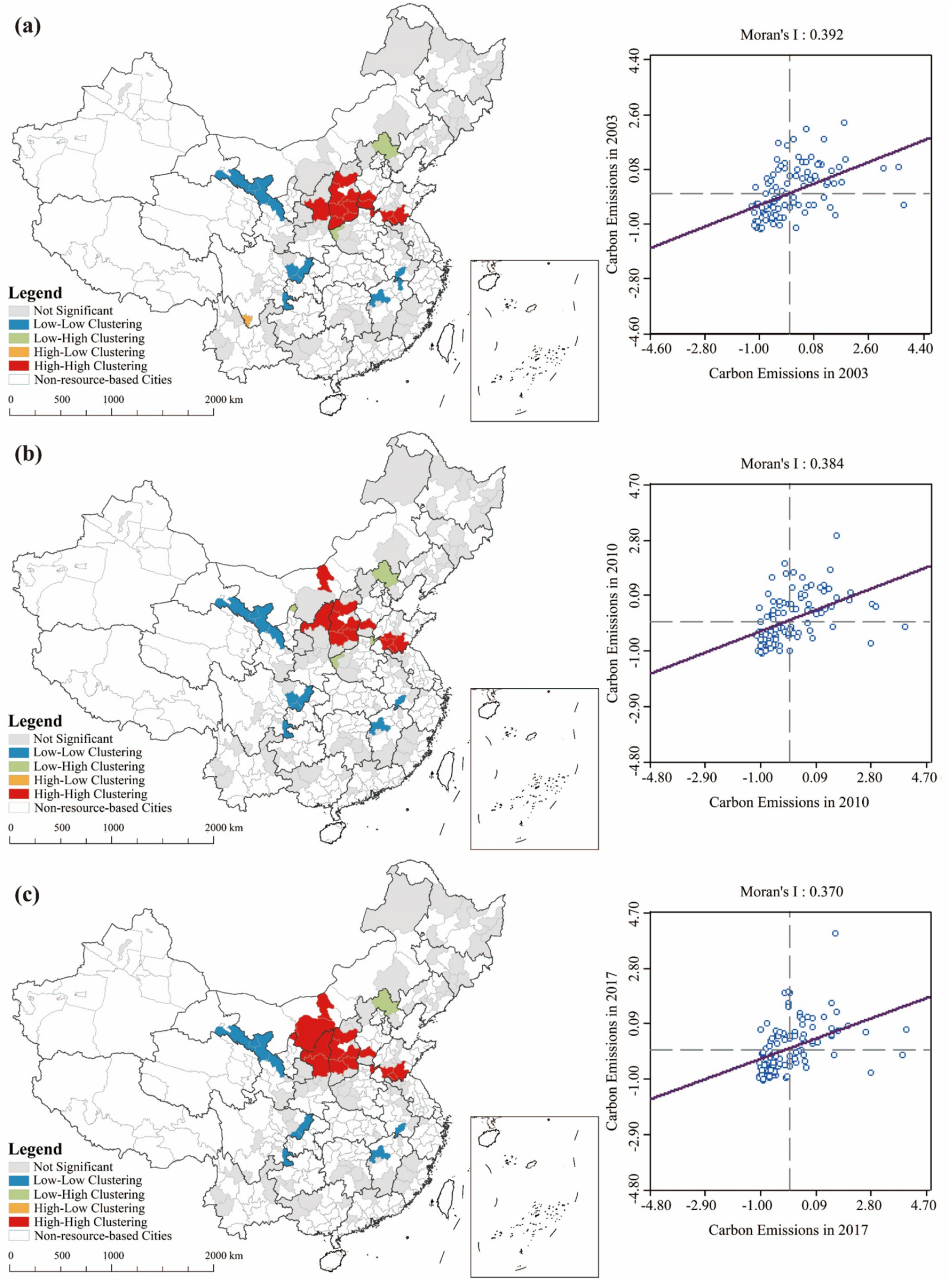
The LISA agglomeration figure and Moran scatter plot of total carbon emissions in Chinese RBCs from 2003 to 2017; (**a**) Result in 2003; (**b**) Result in 2010; (**c**) Result in 2017 (source: GS(2019)1822).

Using local Moran’s $I$ index and Moran scatter plots, this paper tested for spatial correlation of carbon emissions among RBCs to examine further clustering characteristics and trends (see Fig. 8).

During the sample period, a clear spatial autocorrelation existed among RBCs in China, as evident from the global Moran's *I* index of carbon emissions from RBCs. This index exceeded zero between the years 2003 and 2017, indicating high statistical significance. It should be noted that as time progressed, the global Moran's *I* index decreased, from 0.409 in 2003 to 0.389 in 2017, which implies a slight reduction in spatial autocorrelation.

As the global Moran's *I* index can only investigate the presence of spatial autocorrelation from a global perspective and ignores the potential instability of spatial processes, further examination of local autocorrelation is required. We adopted the LISA agglomeration figure and Moran scatter plot to analyze the local autocorrelation characteristics of carbon emissions from RBCs (see Fig. 8). From 2003 to 2017, "low-low" and "high-high" clustering were the most common type of local spatial autocorrelation among RBCs in China. High-value cluster zones were found in the northern part of Shanxi Province, the central and southern parts of Shaanxi and Inner Mongolia Provinces, while low-value cluster zones were scattered in provinces such as Gansu, Sichuan, Jiangxi, and Anhui (see Fig. 8). According to the classification of RBCs based on resource type^66^, it can be found that high-value cluster zones are areas where RBCs with abundant fossil energy resources are concentrated, while low-value cluster zones tend to occur in areas adjacent to RBCs with abundant resources of ferrous and nonferrous metals. Therefore, the dominant resource type and the type of adjacent cities may have a close relationship with the city's carbon emissions, which is one of the main reasons for the occurrence of high-value and low-value cluster zones.

During the period under review, the spatial autocorrelation of carbon emissions from RBCs did not change significantly. Overall, only the number of high-value cluster zones experienced significant changes. High-value cluster zones were concentrated in the vicinity of the three provinces of Shaanxi, Shanxi, and Inner Mongolia, and their number increased over time. This region is the largest energy production base in China. Since 2006, the State Energy Administration of China has approved the construction of multiple large coal production and development bases there. Consequently, coal production in Shaanxi, Shanxi, and Inner Mongolia increased by 121.28% from 2006 to 2017, and total coal production contributed 71.38% of total national output in 2020. Coal production, power generation, and chemical fertilizer production have all increased total carbon emissions in RBCs located in these provinces, creating an expanding area of spatial autocorrelation that is "high-high".

### Analysis of the contributors of carbon emissions in Chinese RBCs

#### Comparative analysis of fitting results

Based on existing research, this article examines the factors influencing carbon emissions in Chinese RBCs from four perspectives: population, economy, industry, and technology (see Table 2). One of the major contributors to the rise of carbon emissions is population growth. By increasing production and consumption, population growth contributes to carbon emissions. It was therefore decided to adopt the permanent population at the end of a year as a representative of the population size^14^. Improvement in socio-economic development level can affect regional carbon emissions by promoting changes in production patterns, technical levels, and consumption concepts. To evaluate the influence of economic factors on carbon emissions, per capita GDP was employed. Industrial development is the main source of carbon emissions due to energy consumption^30^. For RBCs, as resource-based industries are the key support industry for urban development, they are the most outstanding characteristic of RBCs, compared to other cities. Therefore, the proportion of resource-based industries was used to represent the industrial factor. To determine the proportion of resource-based industries, the ratio of mining workers to the total number of workers in the first, second, and third industries was used. Further, enhancing energy efficiency can effectively reduce carbon emissions^67^. Limited by data acquisition at the municipal level, this article referred to existing research^56,68,69^ and selected carbon intensity as the characteristic indicator of carbon abatement technology. In the city with a smaller value, there is a higher level of carbon abatement technology.

**Table 2:** Description of each variable in the model.

Variables		Definition	Unit
Carbon emissions	Total CO_2_ emissions	10^5^t	
Population	Population size	permanent population at the end of a year	Million people
Economy	Per capita GDP	net domestic product (GDP) per inhabitant during the accounting period divided by the total population within the specified area	10 thousand yuan
Industry	Proportion of resource-based industry	the proportion of workers in the mining and extraction industry among the total number of workers in the primary, secondary, and tertiary industries	%
Carbon abatement technology	Carbon intensity	CO_2_ emissions per unit GDP	Tons

As indicated by the spatial autocorrelation analysis discussed earlier, RBCs’ carbon emissions exhibit a distinct spatial clustering pattern, which indicates that the relationship between carbon emissions from RBCs and multiple contributors does not satisfy the requirement for independence between regions outlined in Ordinary Least Squares (OLS). Furthermore, RBCs in China display significant differences in resource endowment, development stage, terrain conditions, and spatial positioning. The degree to which various factors affect carbon emissions may vary greatly among resource-based industries, development stages, and spatial locations in RBCs. Therefore, it is necessary to introduce spatial diversity and spatial dependence to modify the classical linear model. The expression can be obtained as follows:

4 $$\begin{array}{*{20}c} {\begin{array}{*{20}c} {\left( {CO_{2} } \right)_{i} = \propto_{0} \left( {\mu_{i} ,v_{i} } \right) + \beta_{0} \left( {\mu_{i} ,v_{i} } \right)\left( {Pop} \right)_{i} + \beta_{1} \left( {\mu_{i} ,v_{i} } \right)\left( {PGDP} \right)_{i} } \\ { + \beta_{2} \left( {\mu_{i} ,v_{i} } \right)\left( {Res} \right)_{i} + \beta_{3} \left( {\mu_{i} ,v_{i} } \right)\left( {CI} \right)_{i} + \varepsilon_{i} } \\ \end{array} } \\ \end{array}$$

where ${\left({CO}_{2}\right)}_{i}$ is the carbon emissions in city $i$; ${x}_{ik}$ is the independent variable $x_{k}$ in city $i$; $\left( {\mu_{i} ,v_{i} } \right)$ denotes the location of city $i$; $\beta_{k} \left( {\mu_{i} ,v_{i} } \right)$ represents the estimated parameter for independent variable $x_{k}$ at city $i$; $\varepsilon_{i}$ is the random error.

Adaptive spatial kernel regression was used as the basis for this study. The AICc method was used to select a Gaussian model to calculate the optimal bandwidth of the GWR model. A comparison study was conducted on data from different years of RBCs to establish models. Lower AICc values and higher adjusted R^2^ values indicate better performance and accuracy for a model. The results for 2003, 2007, 2012, and 2017 are presented in Table 3. As shown in Table 3, the AICc values and adjusted R^2^ values of the OLS models and the GWR models are relatively high (see Table 3), indicating that various factors provide high explanation power for the carbon emissions of RBCs. Compared to OLS, the GWR model exhibits lower AICc values and higher adjusted R2 values, indicating superior fit quality and model performance, attributed to its consideration of spatial heterogeneity.

**Table 3:** Diagnostic information of OLS and GWR.

Year	Model	AICc	R^2^	Adj. R^2^
2003	OLS	634.56	0.74	0.73
	GWR	620.92	0.79	0.76
2007	OLS	753.40	0.76	0.75
	GWR	735.35	0.81	0.79
2012	OLS	853.72	0.75	0.74
	GWR	839.22	0.80	0.77
2017	OLS	854.20	0.73	0.72
	GWR	837.12	0.79	0.76

#### Analysis of the relationship between population size and carbon emissions

**Figure 9:**
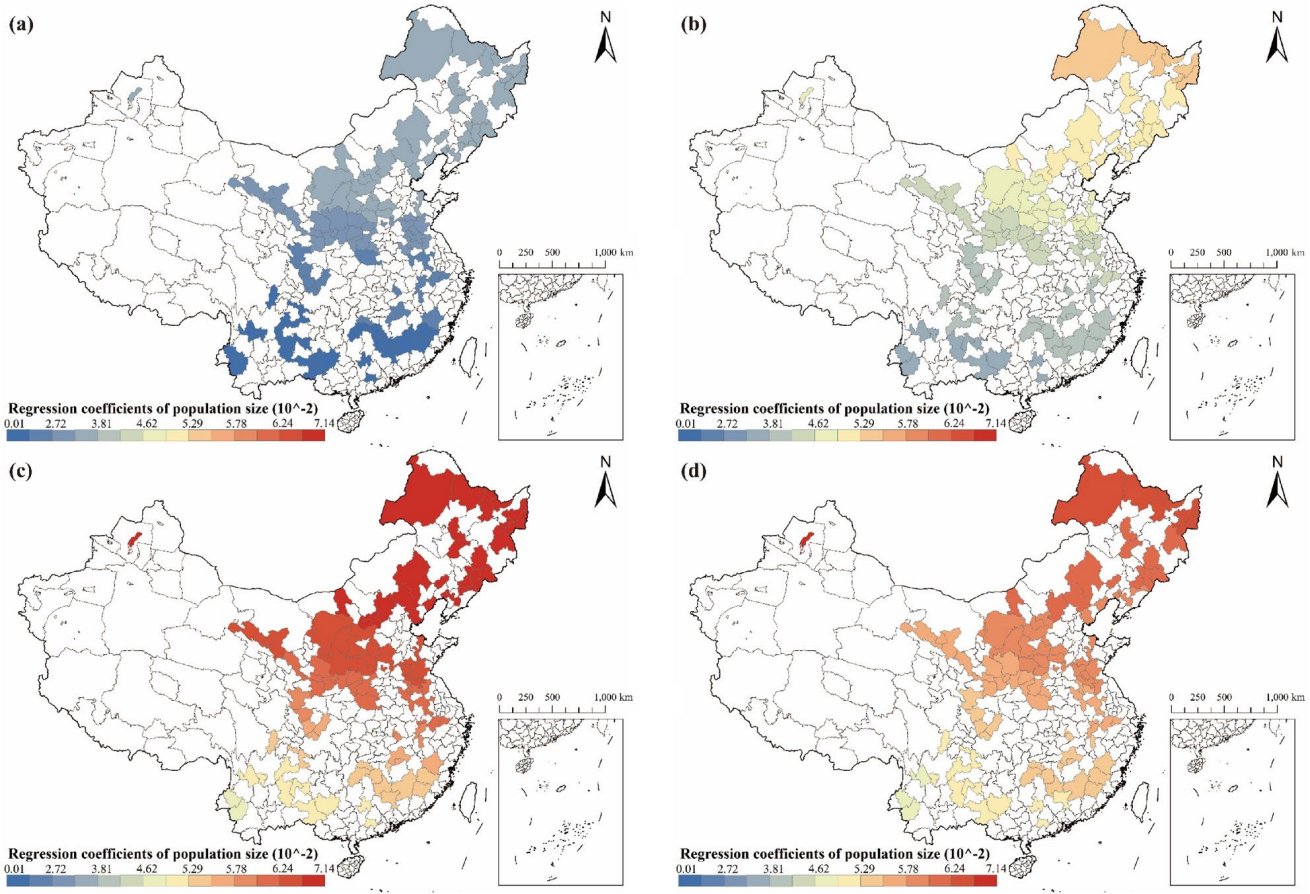
Regression coefficients of population size in RBCs: (**a**) Result in 2003; (**b**) Result in 2007; (**c**) Result in 2012; (**d**) Result in 2017 (source: GS(2019)1822).

Significant spatial differences are evident in the effects of population size on carbon emissions in RBCs (see Fig. 9). The positive correlation between population size and carbon emissions, as depicted in Fig. 9, suggests that an increase in population size is associated with a rise in carbon emissions. During the study period, RBCs exhibited a pattern where the magnitude of the population size regression coefficient initially increased and then decreased, suggesting that the sensitivity of carbon emissions to changes in population size increased and then decreased over time. Concerning spatial distribution, population size regression coefficients showed a trend toward an increase in spatial distribution from south to north. Moreover, population size impact coefficients in RBCs displayed more rapid variations in northern regions over time, indicating heightened sensitivity to population changes in these cities.

#### Analysis of the relationship between the level of economic development and carbon emissions

**Figure 10:**
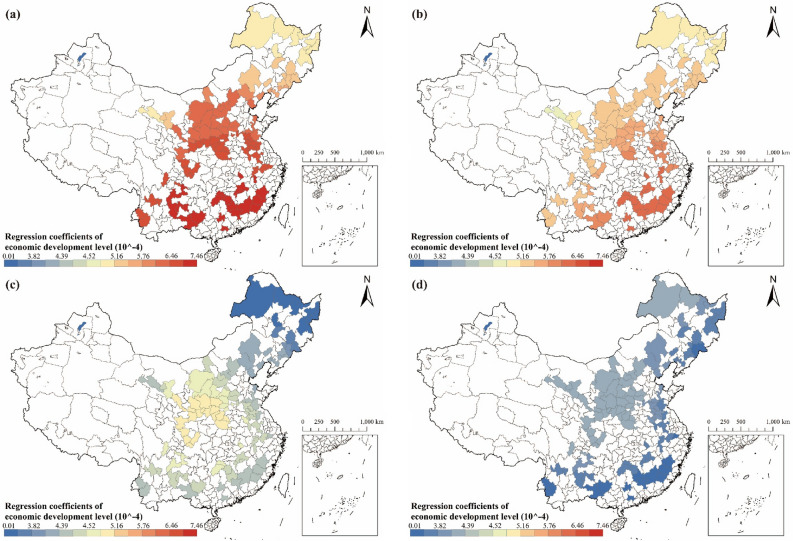
Regression coefficients of economic development level in RBCs: (**a**) Result in 2003; (**b**) Result in 2007; (**c**) Result in 2012; (**d**) Result in 2017 (source: GS(2019)1822).

Economic development has significantly increased carbon emissions in RBCs, with a spatially differentiated impact degree (see Fig. 10). Except for RBCs in the northeast region, the regression coefficient values for the economic development level in each RBC decreased gradually over time. This trend indicates that while economic growth in RBCs continued to be associated with an increase in carbon emissions, the amount of carbon dioxide produced per unit of GDP per capita has decreased in recent years. There was a gradual shift away from energy-intensive or highly polluting production patterns in RBCs. Since 2012, the influence of economic development on carbon emissions has increased in RBCs situated in the northeast region. It can be concluded that the carbon emissions generated by each unit of per capita GDP increased, suggesting that RBCs in this region did not have a favorable transformation effect.

In 2003, the regression coefficient for economic development levels followed a latitudinal pattern, increasing from north to south. By 2007, this pattern shifted to 'higher along the coast and lower inland,' and further evolved to 'lower along the coast and higher inland' by 2012 (see Fig. 10). This transformation can be attributed to the declining impact of economic growth on carbon emissions in the east coastal regions, contrasting with a slower reduction observed in central China compared to other areas. It suggests that RBCs in central China have more rapidly shifted away from the growth model that prioritizes profit over the environment. Additionally, studies indicate regional variations in China's circular economy development, showing a gradual decline in the East, Central, and West regions. As anticipated, the development level of the circular economy in the East was higher^70,71^, providing further confirmation of the above hypothesis. China's industrial transfer policy, initiated in 2010 with the 'Guiding Opinions of the State Council on Undertaking Industrial Transfer in the Central and Western Regions,' accelerated the shift of industries from the eastern coastal regions to the central and western regions. Leveraging abundant resources, low factor costs, and substantial market potential, the central and western regions actively embraced industrial transfers. Research suggests that outward industrial transfer negatively impacts carbon emissions in the transferring regions^72^. Conversely, recipient regions experience an increase in provincial carbon emissions. While acknowledging potential carbon reduction in the eastern regions due to technological advancements and enhanced environmental awareness accompanying industrial transfers, a comparative analysis of carbon emissions in different geographic and industrial transfer zones reveals an overall increase in carbon emissions in recipient areas^72^. Therefore, China's industrial transfer policy may be another key factor contributing to the significant reduction in the impact of the economic development level on carbon emissions in the eastern coastal regions in 2012.

#### Analysis of the relationship between carbon abatement technology and carbon emissions

**Figure 11:**
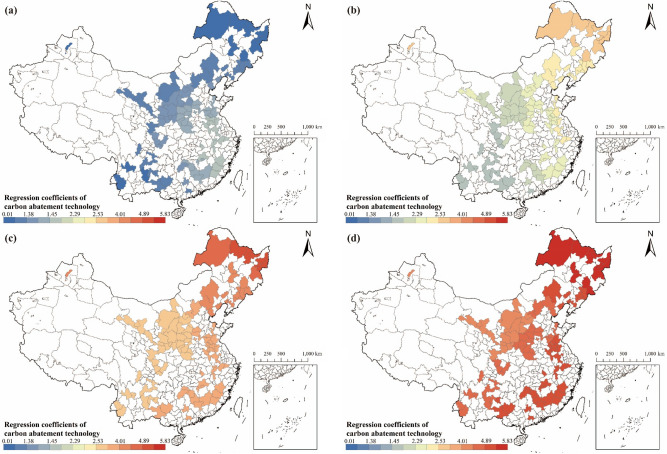
Regression coefficients of carbon abatement technology in RBCs: (**a**) Result in 2003; (**b**) Result in 2007; (**c**) Result in 2012; (**d**) Result in 2017 (source: GS(2019)1822).

Carbon abatement technology emerges as the predominant factor influencing carbon emissions in RBCs, exhibiting a negative impact on emissions (see Fig. 11). The absolute values of the regression coefficients of carbon abatement technology proficiency in each RBC are much higher than those of other factors, showcasing a growing trend over time. This underscores the increasing significance of carbon abatement technology in shaping RBCs' carbon emissions, with its impact intensifying over the years. In addition, it should be noted that the influence of carbon abatement technology on carbon emissions in RBCs is spatially heterogeneous, exhibiting a stable spatial pattern, with carbon emissions being "high in coastal areas and low in inland areas". The emergence of such a spatial pattern is predominantly a result of the unbalanced and inadequate development of carbon abatement technology capabilities in different regions of China. Especially in high-tech areas where advanced carbon abatement technology is used, technology plays a more significant role in carbon reduction. Research confirms that China's coastal provinces, driven by higher economic development, greater openness, and substantial investment in research and development, have matured their carbon abatement technology more effectively^73^. Consequently, the development of technology in coastal RBCs has proven more effective at reducing carbon than those in inland RBCs.

Specifically, from 2003 to 2017, the significance of carbon abatement technology in RBCs in the southeastern and northeastern regions of China increased rapidly, and even more rapidly than other RBCs. This means that during this period, the advancement of carbon abatement technology in these regions increased more rapidly. Environmental regulations are of great significance in promoting environment-biased and energy-biased technological progress^74^. Since the dawn of the twenty-first century, environmental regulations in the Northeastern region have entered an optimization stage. Strategic initiatives, such as the Strategy to Revitalize the Old Industrial Bases in China's Northeast, revitalized the old industrial base and provided a new historical mission to the region. During this stage, there has been continuous improvement in environmental regulations in the Northeastern region, with a significant increase in the number of regulations and comprehensive coverage of various fields related to environmental pollution and ecological destruction. According to statistics, from 2000 to 2018, the number of environmental regulations and policies adopted by the Heilongjiang, Jilin, and Liaoning provinces respectively accounted for 96.0%, 92.0%, and 95.3% of the total number of environmental regulations and policies adopted since 1972^75^. The optimization of environmental regulations was driving rapid progress in carbon abatement technology in RBCs in the northeast region, allowing these technologies to play a more important role.

#### Analysis of the relationship between the proportion of resource-based industries and carbon emissions

**Figure 12:**
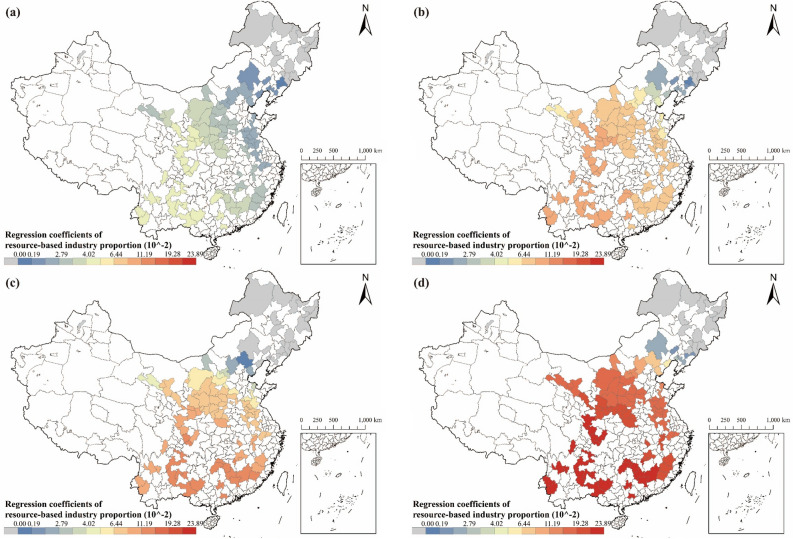
Regression coefficients of resource-based industry proportion in RBCs: (**a**) Result in 2003; (**b**) Result in 2007; (**c**) Result in 2012; (**d**) Result in 2017 (source: GS(2019)1822).

In comparison with other factors, the proportion of resource-based industries have the second greatest impact on carbon emissions. There is spatial heterogeneity in the effects of the proportion of resource-based industries on carbon emissions in RBCs (see Fig. 12). During the period 2003 to 2007, the proportion of resource-based industries impacted carbon emissions with a spatial pattern of "low in the coastland and high in the hinterland", which is in general aligned with the spatial characteristic of economic development in China. Over the last few decades, the eastern coast of China has been a leading force in reform and opening up, fostering strong connections with other nations, and experiencing relatively high economic development. As a result, the spatial distribution in the extent of the impact of resource-based industries, shown in Fig. 12, is primarily a result of the more diverse industrial structure of RBCs along the eastern coast, which has a lower proportion of resource-based industries compared to regions inland. Thus, in the eastern coastal RBCs, the share of resource-based industries had a lesser effect on carbon emissions. Moreover, since 2012, the effect of the proportion of resource-based industries on carbon emissions in RBCs has shifted to a spatial characteristic with "low in the northeast and high in the southwest". The reason for the occurrence of this transition is the slower growth rate of resource-based industries in RBCs in southeastern coastal areas than in other RBCs. As previously mentioned, renewable energy technologies, energy-saving technology in energy-intensive industries, and residential energy technologies in the southeastern coastal areas of China are more advanced and rapidly developing^72^. Therefore, despite the expansion of resource-based industries in southeastern coastal RBCs, the impact of these industries on carbon emissions was slowing down due to the progress of energy technologies.

#### Summary

In summary, the factors of population size, economic development level, technical capabilities for carbon reduction, and the proportion of resource-based industries all interact and synergistically affect carbon emissions in RBCs. Among these factors, technical capability for carbon reduction is the dominant factor affecting carbon emissions in RBCs, followed by the proportion of resource-based industries. The economic development level has the weakest impact on carbon emissions. This illustrates how energy and carbon abatement technologies are becoming increasingly important in the reduction of carbon emissions. Despite their limited impact, there is a gradual shift away from energy-intensive or highly polluting production patterns in RBCs.

Spatially, a significant spatial difference was observed in the degree to which each factor affected carbon emissions, during the study period. The impact of population size increased from south to north, while the impact of technical capabilities for carbon reduction showed a stable "coastal high—inland low" spatial pattern. Influenced by the development of RBCs in the southeastern coastal region, there were changes in the spatial pattern of the effect of the level of economic development, the proportion of resource-based industries, and energy consumption on carbon emissions: the effect of the level of economic development on carbon emissions has shifted spatially from the "south high—north low" pattern to the "west high—east low" and "northwest high—southeast low" pattern; the effect of the proportion of resource-based industries shifted from "coastal low—inland high" to "northeast low—southwestern high". Therefore, over the study period, the alternations in the spatial differentiation of the effect of each factor on carbon emissions can be predominantly attributable to the development of RBCs in the southern coastal region. In summary, during the study period, RBCs located in the southeastern coastal region of China made more significant advances in the innovation of energy and carbon abatement technologies and were able to overcome the growth model that sacrifices the environment for profit more quickly, but the effectiveness of these technologies remained limited and needs to be further improved.

## Conclusions

### Main findings

In this article, we first examined the spatial pattern of carbon emissions in RBCs along with their evolving characteristics over time. Spatial heterogeneity and spatial dependence were then introduced as a moderator of the classic linear model to explore the contributors of carbon emissions in RBCs based on four perspectives, namely population, economy, industry, and technology. Here are the primary findings.

1. Between 2003 and 2017, it is estimated that the total carbon emissions from RBCs amounted to around 30% of the total national output, and showed a "swift-then-slack" upward trend with a breakpoint in 2011. In this period, a slight decrease in the carbon emissions ratio was observed in the central and Northeastern regions, whereas the western regions experienced a significant increase. Growth-oriented RBCs displayed a higher carbon emissions ratio year after year, whereas mature RBCs exhibited a decrease in carbon emissions ratio. Although RBCs’ carbon emissions stabilized in 2011, their emissions have not peaked yet and there is a possibility of a further increase in the future. Carbon emissions from RBCs showed a higher growth rate from 2003 to 2013, compared to non-resource-based cities, and a lower rate between 2014 and 2017. The breakpoint in 2014 may be related to the issue of the Sustainable Development Plan for National Resource-Based Cities (2013–2020).
2. The carbon emissions from RBCs displayed a stable "high for northeast and low for southeast" pattern in space, with high spatial autocorrelation. The difference in carbon emissions between cities in RBCs was gradually increasing, but on average, the gap between RBCs was smaller than that between non-resource-based cities and the national average. Furthermore, further analysis utilizing a spatial autocorrelation model reveals that the spatial autocorrelation of carbon emissions in RBCs was highly pronounced, and the local spatial autocorrelation pattern was relatively stable. High-value cluster zones were mainly concentrated in the northern part of Shaanxi, Shanxi, and Inner Mongolia, while low-value cluster zones were scattered in provinces such as Gansu, Sichuan, Jiangxi, and Anhui. Accordingly, RBCs in China's Inner Mongolia, Shaanxi, and Shanxi regions, as well as those in the northeast of China, were the major contributors to carbon emissions. Besides, the high-value cluster zones were concentrated areas where RBCs with abundant fossil fuels are located, while the low-value cluster zones tended to occur in areas adjacent to RBCs with abundant ferrous and nonferrous metals. It appears that the type of primary resources and the type of adjacent cities directly influence the carbon emissions of the city, which may explain the emergence of high-value cluster zones and low-value cluster zones.
3. Population size, the level of economic development, carbon abatement technology, and the proportion of resource-based industries all interact and have synergistic effects on carbon emissions in RBCs. Carbon abatement technology is the key contributing factor to RBCs’ carbon emissions, followed by the proportion of resource-based industries. Among the contributors to carbon emissions, the level of economic development is of the least significance. There is no doubt that carbon abatement technology is becoming progressively vital in reducing carbon emissions. Even though its impact is limited, RBCs are gradually overcoming the inefficient production model characterized by “energy intensive or highly polluting”. Therefore, innovation in energy and carbon abatement technology should still be the focus of future work in RBCs.
4. There are spatial differences in the extent to which factors influence carbon emissions. The influence of population size increased from south to north, while the impact of carbon abatement technology showed a stable “high on the coast and low inland” spatial pattern. It is worth noting that influenced by the advancement of RBCs situated in the southeastern coastal region, the spatial patterns of the effect of the level of economic development and the proportion of resource-based industries on carbon emissions changed over time: the effect of the level of economic development on carbon emissions gradually changed from "south high—north low" to " higher along the coast and lower inland " and " lower along the coast and higher inland " in space; the effect of the proportion of resource-based industries shifted from "coastal low—inland high" to "northeast low—southwestern high". Accordingly, the development of RBCs located in southeast and eastern coastal regions of China is the primary cause of the changing spatial patterns of the influence of various factors. During the study period, rapid progress was made in carbon abatement technology of RBCs in the southeast and eastern coastal regions, enabling these cities to get rid of the growth model that sacrifices the environment more quickly, compared to RBCs in other regions. However, the role of technology was limited and needs further improvement

In the current debates on climate change mitigation policies, resource-rich regions have long been ignored. In terms of the scope of current research on carbon emissions and its contributors, it primarily focuses on the global, an economic and political community, a certain country, a certain province, or a city cluster, with research units ranging from countries and regions to provinces, cities, and counties. However, there has been a notable gap in addressing the distinctions between resource-rich and resource-poor regions. In this article, we take RBCs, which have a special socio-economic structure, as the research area, and explore the sensitivity of carbon emissions of different RBCs to different influencing factors and their causes. This can help to better understand resource-rich regions and the obstacles they must overcome in reducing carbon emissions and can support a comprehensive understanding of the basic challenges they face when striving to reduce carbon emissions.

### Policy implications

Based on the aforementioned conclusions, this paper suggests the following measures for reducing carbon emissions in RBCs.

1. Emphasize technological innovation and promote carbon emission reduction with multiple initiatives. It is noteworthy that technology innovation can greatly decrease carbon emissions, and its fluence on carbon emissions surpass other factors such as industry and population. This suggests that future efforts to reduce carbon emissions should prioritize the promotion of technological innovation, the elimination of outdated production capacity, the restructuring of energy systems, and the promotion of energy efficiency. Therefore, it is advisable to implement low-carbon technological innovations, with a specific focus on fostering the green development of fossil energy, promoting low-carbon utilization, and reducing pollution. These efforts should be complemented by strengthening energy-saving and emission reduction systems and mechanisms, as well as initiating the reconstruction of the energy structure and the transfer of high-energy-consuming industries. These collective measures aim to effectively advance the cause of energy conservation and emission reduction.
2. Prioritize Central China's Resource-Based Cities (RBCs) in upcoming carbon reduction initiatives and expedite the phase-out of obsolete production capacity and outdated technology in these regions. Given their significant contribution to emissions among RBCs, it is imperative to prioritize the carbon emission work of RBCs in Central China. Future endeavours should encompass proactive measures, including actively promoting the application of energy efficiency and emission reduction technologies, reconstructing the energy consumption structure, eliminating the outdated production capacity and promoting renewable energy.
3. Harness the abundant renewable energy in Western RBCs to take over energy-intensive industries from Eastern and Central China. Carbon emissions from RBCs in Western China has been increasing annually, with higher accelerating rate compared to national average. Therefore, it is necessary to place increased emphasis on carbon emission reduction efforts in these regions. However, being an underdeveloped area in China, the imperative for fostering economic development in Western China makes inheriting high-consumption and high-emission industries from Eastern and Central China inevitable for RBCs in Western China. A positive aspect is the abundant renewable energy resources, such as wind and solar power, that Western RBCs in China are endowed with. This endowment enables Western RBCs to transition from conventional energy sources like coal and gas to renewable alternatives, including wind power, solar photovoltaic, solar thermal, and hydropower. This shift aligns with the dual objectives of fostering economic development and reducing carbon emissions.

## Data Availability

The datasets generated during and/or analysed during the current study are available from the corresponding author on reasonable request.

## References

[1] Leal Filho W, Tuladhar L, Li C, Balogun A-LB, Kovaleva M, Abubakar IR, Azadi H, Donkor FKK (2023). Climate change and extremes: Implications on city livability and associated health risks across the globe. Int. J. Clim. Change Strateg. Manag..

[2] Liu Z, Deng Z, He G, Wang H, Zhang X, Lin J, Qi Y, Liang X (2021). Challenges and opportunities for carbon neutrality in China. Nat. Rev. Earth Environ..

[3] Xiao R, Tan G, Huang B, Li J, Luo Y (2022). Pathways to sustainable development: Regional integration and carbon emissions in China. Energy Rep..

[4] Yu J, Li J, Zhang W (2019). Identification and classification of resource-based cities in China. J. Geog. Sci..

[5] Lu S, Zhang W (2020). The identification of spatial evolution stage of resource-based cities and its development characteristics. Acta Geographica Sinica.

[6] Li H, Long R, Chen H (2013). Economic transition policies in Chinese resource-based cities: An overview of government efforts. Energy Policy.

[7] Liu S, Jiang G, Chang L, Huang C (2023). Construction and simulation of high-quality development of China’s resource-based cities driven by innovation based on system dynamics. Int. J. Environ. Res. Public Health.

[8] Xie W, Chapman A, Yan T (2023). Do environmental regulations facilitate a low-carbon transformation in China’s resource-based cities?. Int. J. Environ. Res. Public Health.

[9] Friedrichs J, Inderwildi OR (2013). The carbon curse: Are fuel rich countries doomed to high CO_2_ intensities?. Energy Policy.

[10] Wang X, Wu Ji, Bai B, Wang Z (2020). Spatial differentiation and driving factors of CO_2_ emissions: Analysis based on 198 cities at prefecture level and above in China. Econom. Geogr..

[11] Engelman, R. *Stabilizing the Atmosphere: Population, Consumption and Greenhouse Gases* (1994). 10.13140/RG.2.2.26843.31522

[12] Lawal IM (2019). Impact of population growth on Carbon Dioxide (CO_2_) emission: Empirical evidence from Nigeria. Jurnal Perspektif Pembiayaan Dan Pembangunan Daerah.

[13] Xiang H, Zeng X, Han H, An X (2023). Impact of population aging on carbon emissions in China: An empirical study based on a kaya model. Int. J. Environ. Res. Public Health.

[14] Satterthwaite D (2009). The implications of population growth and urbanization for climate change. Environ. Urban..

[15] Li X, Ullah S (2022). Caring for the environment: How CO_2_ emissions respond to human capital in BRICS economies?. Environ. Sci. Pollut. Res..

[16] Yin Y, Xiong X, Ullah S, Sohail S (2021). Examining the asymmetric socioeconomic determinants of CO_2_ emissions in China: Challenges and policy implications. Environ. Sci. Pollut. Res..

[17] Li X, Ozturk I, Majeed MT, Hafeez M, Ullah S (2022). Considering the asymmetric effect of financial deepening on environmental quality in BRICS economies: Policy options for the green economy. J. Clean. Prod..

[18] Grossman, G. M., & Krueger, A. B. *Environmental Impacts of a North American Free Trade Agreement* (Working Paper No. 3914). National Bureau of Economic Research (1991). 10.3386/w3914

[19] Panayotou, T. Empirical tests and policy analysis of environmental degradation at different stages of economic development. *ILO Working Papers*, Article 992927783402676 (1993). https://ideas.repec.org//p/ilo/ilowps/992927783402676.html

[20] Albino V, Ardito L, Dangelico RM, Messeni Petruzzelli A (2014). Understanding the development trends of low-carbon energy technologies: A patent analysis. Appl. Energy.

[21] Cheng Z, Li L, Liu J (2018). Industrial structure, technical progress and carbon intensity in China’s provinces. Renew. Sustain. Energy Rev..

[22] Shao X, Zhong Y, Li Y, Altuntaş M (2021). Does environmental and renewable energy R&D help to achieve carbon neutrality target? A case of the US economy. J. Environ. Manage..

[23] Elsheikh AH, Shanmugan S, Sathyamurthy R, Thakur AK, Issa M, Panchal H, Muthuramalingam T, Kumar R, Sharifpur M (2022). Low-cost bilayered structure for improving the performance of solar stills: Performance/cost analysis and water yield prediction using machine learning. Sustain. Energy Technol. Assess..

[24] Elsheikh AH, Panchal H, Shanmugan S, Muthuramalingam T, El-Kassas A, M., Ramesh, B.  (2022). Recent progress in wood-plastic composites: Pre-processing treatments, manufacturing techniques, recyclability and eco-friendly assessment. Clean. Eng. Technol..

[25] Shirong, G., Jingli, F., Shuqin, L., Mei, S., Yujiao, X., Bing, W., Teng, T. Low carbon modern coal-based energy technology system and development strategy. *Journal of China Coal Society,* 1–26 (2023). 10.13225/j.cnki.jccs.2023.1773.

[26] Na Z, Qianyu M, Jiawei D, Xiaojun Z (2024). Impact of the rise of new energy on China's new energy industry strategy. China Soft Sci..

[27] Braungardt S, Elsland R, Eichhammer W (2016). The environmental impact of eco-innovations: The case of EU residential electricity use. Environ. Econ. Policy Stud..

[28] Cai A, Zheng S, Cai L, Yang H, Comite U (2021). How does green technology innovation affect carbon emissions? A spatial econometric analysis of China’s provincial panel data. Front. Environ. Sci..

[29] Abdouli M, Hammami S (2017). The impact of FDI inflows and environmental quality on economic growth: An empirical study for the MENA countries. J. Knowl. Econ..

[30] Naminse E, Zhuang J (2018). Economic growth, energy intensity, and carbon dioxide emissions in China. Polish J. Environ. Stud..

[31] Solaymani S (2022). CO_2_ emissions and the transport sector in Malaysia. Front. Environ. Sci..

[32] Kozlowski, A., Bardecki, M., & Searcy, C. *Environmental impacts in the fashion industry: A life-cycle and stakeholder framework*. *Journal of Corporate Citizenship*, 15–34 (2012).

[33] Lu K, Wang H (2019). Estimation of building’s life cycle carbon emissions based on life cycle assessment and building information modeling: A case study of a hospital building in China. J. Geosci. Environ. Protect..

[34] Chen J, Gao M, Cheng S, Hou W, Song M, Liu X, Liu Y, Shan Y (2020). County-level CO_2_ emissions and sequestration in China during 1997–2017. Sci. Data.

[35] Batmunkh A (2022). Carbon footprint of the most popular social media platforms. Sustainability.

[36] Shi M, Wang Y, Zhang Z, Zhou X (2012). Regional carbon footprint and interregional transfer of carbon emissions in China. Acta Geogr. Sin..

[37] Wang S, Huang Y, Zhou Y (2019). Spatial spillover effect and driving forces of carbon emission intensity at the city level in China. Acta Geogr. Sin..

[38] She X, He Q, Yang X, Sun J, Yuan F, Wang Y (2019). Evaluation of carbon emission performance and estimation of CO_2_ abatement costs for provinces of China: A non-parametric distance function approach. Open J. Soc. Sci..

[39] Zhang W, Liu X, Wang D, Zhou J (2022). Digital economy and carbon emission performance: Evidence at China’s city level. Energy Policy.

[40] Kim J, Lim H, Jo H-H (2020). Do aging and low fertility reduce carbon emissions in Korea? Evidence from IPAT augmented EKC analysis. Int. J. Environ. Res. Public Health.

[41] Liu Q, Wu S, Lei Y, Li S, Li L (2021). Exploring spatial characteristics of city-level CO_2_ emissions in China and their influencing factors from global and local perspectives. Sci. Total Environ..

[42] Wang S, Liu X (2017). China’s city-level energy-related CO_2_ emissions: Spatiotemporal patterns and driving forces. Appl. Energy.

[43] Gu H, Liu Y, Xia H, Tan X, Zeng Y, Zhao X (2023). Spatiotemporal dynamic evolution and its driving mechanism of carbon emissions in hunan province in the last 20 years. Int. J. Environ. Res. Public Health.

[44] Akimoto H, Narita H (1994). Distribution of SO_2_, NOx and CO_2_ emissions from fuel combustion and industrial activities in Asia with 1° × 1° resolution. Atmos. Environ..

[45] Duro JA, Teixidó-Figueras J, Padilla E (2016). Empirics of the international inequality in CO_2_ emissions intensity: Explanatory factors according to complementary decomposition methodologies. Environ. Resource Econ..

[46] Zhang L, Huang Y, Li Y, Cheng X (2010). An investigation on spatial changing pattern of CO_2_ emissions in China. Resources Science.

[47] Zhao Y, Huang X, Zhong T, Peng J (2011). Spatial pattern evolution of carbon emission intensity from energy consumption in China. Environ. Sci. (CNKI).

[48] Yue C, Hu X, He C, Zhu J, Wang S, Fang J (2010). Provincial carbon emissions and carbon intensity in China from 1995 to 2007 (Carbon Emissions and Social Development, III). Acta Scientiarum Naturalium Universitatis Pekinensis (CNKI).

[49] Clarke-Sather A, Qu J, Wang Q, Zeng J, Li Y (2011). Carbon inequality at the sub-national scale: A case study of provincial-level inequality in CO_2_ emissions in China 1997–2007. Energy Policy.

[50] Jiang W, Liu W, Liu Z, Han M (2020). Inequality and driving forces of energy-related CO_2_ emissions intensity in China. Prog. Geogr..

[51] Wang S, Fang C, Wang Y (2016). Spatiotemporal variations of energy-related CO_2_ emissions in China and its influencing factors: An empirical analysis based on provincial panel data. Renew. Sustain. Energy Rev..

[52] Yan Y, Wang Z, Wu L, Liu C (2016). Analysis of the determinants of carbon emission intensity on regional differences. Acta Scientiae Circumstantiae.

[53] Zeng X, Qiu R, Lin D, Hou X, Zhang L, Hu X (2020). Spatio-temporal heterogeneity of transportation carbon emissions and its influencing factors in China. China Environ. Sci..

[54] Gao C, Liu X, Li Z, Zhang Y, Yu G, Su Q, Tian Y (2016). Spatiotemporal dynamics of carbon emissions by energy consumption in China From 1995 to 2014. Prog. Geogr..

[55] Wang S, Su Y, Zhao Y (2018). Regional inequality, spatial spillover effects, and the factors influencing city-level energy-related carbon emissions in China. Acta Geogr. Sin..

[56] Wang S, Xie Z, Wang Z (2021). The spatiotemporal pattern evolution and influencing factors of CO_2_ emissions at the county level of China. Acta Geogr. Sin..

[57] Xv G, Liu Z, Jiang Z (2006). Decomposition model and empirical study of carbon emissions for China, 1995–2000. China Popul. Resour. Environ..

[58] Sadorsky P (2014). The effect of urbanization on CO_2_ emissions in emerging economies. Energy Econom..

[59] Chontanawat J (2019). Driving forces of energy-related CO_2_ emissions based on expanded IPAT decomposition analysis: Evidence from ASEAN and four selected countries. Energies.

[60] Cheng C, Ren X, Dong K, Dong X, Zhen W (2020). How does technological innovation mitigate CO_2_ emissions in OECD countries? Heterogeneous analysis using panel quantile regression. J. Environ. Manage..

[61] Lantz V, Feng Q (2006). Assessing income, population, and technology impacts on CO_2_ emissions in Canada: Where’s the EKC?. Ecol. Econ..

[62] Cheng Y, Wang Z, Zhang S, Ye X, Jiang H (2013). Spatial econometric analysis of carbon emission intensity and its driving factors from energy consumption in China. Acta Geogr. Sin..

[63] Li J, Huang X, Wu C, Zhou Y, Xv G (2015). Analysis of spatial heterogeneity impact factors on carbon emissions in China. Econom. Geogr. (CNKI).

[64] Zhang C, Lin Y (2012). Panel estimation for urbanization, energy consumption and CO_2_ emissions: A regional analysis in China. Energy Policy.

[65] Wang Q, Wu S, Zeng Y, Wu B (2016). Exploring the relationship between urbanization, energy consumption, and CO_2_ emissions in different provinces of China. Renew. Sustain. Energy Rev..

[66] Li B, Zhang W (2016). A study on total factor energy efficiency and its difference in resource-based cities in china with consideration of environmental constraints. J. Natl. Res..

[67] Huang R, Wang Z, Ding G, Gong Y, Liu C (2016). Trend prediction and analysis of influencing factors of carbon emissions from energy consumption in Jiangsu province based on STIRPAT model. Geogr. Res..

[68] Liu Q, Li Q, Zheng X (2017). The prediction of carbon dioxide emissions in Chongqing based on fossil fuel combustion. Acta Scientiae Circumstantiae.

[69] Wang Y, Bi Y, Wang E (2017). Scene prediction of carbon emission peak and emission reduction potential estimation in Chinese industry. China Popul. Resour. Environ..

[70] Bai L, Bai Y, Xue Y, Chen F (2007). Circular economy beforehand evaluation of provinces and regional diversity. Scientia Geographica Sinica.

[71] Sun J, Li G, Wang Z (2019). Technology heterogeneity and efficiency of China’s circular economic systems: A game meta-frontier DEA approach. Resour. Conserv. Recycl..

[72] Wang Y (2022). Effects of Industrial Transfer on Provincial Carbon Emission from the Perspective of Spatial Correlation Network (Unpublished doctoral dissertation).

[73] Wang B, Wang Z (2018). Heterogeneity evaluation of China’s provincial energy technology based on large-scale technical text data mining. J. Clean. Prod..

[74] Zhou X, Xia M, Zhang T, Du J (2020). Energy- and environment-biased technological progress induced by different types of environmental regulations in China. Sustainability.

[75] Xia, X. *Study on the Influence of Environmental Regulation on Economic Growth in Northeast China* [Doctor, Jilin University] (2019). https://kns.cnki.net/kcms2/article/abstract?v=3uoqIhG8C447WN1SO36whLpCgh0R0Z-iDdIt-WSAdV5IJ_Uy2HKRAf1CKACrB0aqy-Q5AcwnhplVD4HbIBoVKs8F-va-IhOi&uniplatform=NZKPT

