# Author Correction: Determinants and their spatial heterogeneity of carbon emissions in resource-based cities, China

**DOI:** 10.1038/s41598-024-60552-2

**Published:** 2024-04-29

**Authors:** Chenchen Guo, Jianhui Yu

**Affiliations:** 1 Institute of Geographic Sciences and Natural Resources Research, CAS, Beijing, 100101 China; 2 College of Resources and Environment, University of Chinese Academy of Sciences, Beijing, 100049 China; 3 CAS Key Laboratory of Regional Sustainable Development Modeling, Beijing, China

Correction to: *Scientific Reports* 10.1038/s41598-024-56434-2, published online 11 March 2024

The original version of this Article contained an error in Affiliation 2, which was incorrectly given as ‘University of Chinese Academy of Sciences, Beijing, 100049, China. The correct affiliation is listed below:

College of Resources and Environment, University of Chinese Academy of Sciences, Beijing, 100049, China.

Additionally, the Acknowledgments section was omitted. The Acknowledgments section now reads:

“Our research received support from the National Science Foundation of China [Grant Number 42171290].”

The original Article has been corrected.

